# Data Exchange Interoperability in IoT Ecosystem for Smart Parking and EV Charging

**DOI:** 10.3390/s18124404

**Published:** 2018-12-13

**Authors:** Anastasiia Karpenko, Tuomas Kinnunen, Manik Madhikermi, Jeremy Robert, Kary Främling, Bhargav Dave, Antti Nurminen

**Affiliations:** 1 Department of Computer Science, Aalto University, 02150 Espoo, Finland; tuomas.kinnunen@aalto.fi (T.K.); Manik.Madhikermi@aalto.fi (M.M.); Kary.Framling@aalto.fi (K.F.); Bhargav.Dave@aalto.fi (B.D.); antti.nurminen@aalto.fi (A.N.); 2 SnT—Interdisciplinary Centre for Security, Reliability and Trust, University of Luxembourg, Luxembourg L-1359, Luxembourg; jeremy.robert@uni.lu

**Keywords:** Internet of Things, smart city, interoperability, data exchange, messaging standards, ecosystem, O-MI, O-DF

## Abstract

Many domains are trying to integrate with the Internet of Things (IoT) ecosystem, such as public administrations starting smart city initiatives all over the world. Cities are becoming smart in many ways: smart mobility, smart buildings, smart environment and so on. However, the problem of non-interoperability in the IoT hinders the seamless communication between all kinds of IoT devices. Different domain specific IoT applications use different interoperability standards. These standards are usually not interoperable with each other. IoT applications and ecosystems therefore tend to use a vertical communication model that does not allow data sharing horizontally across different IoT ecosystems. In 2014, The Open Group published two domain-independent IoT messaging standards, O-MI and O-DF, aiming to solve the interoperability problem. In this article we describe the practical use of O-MI/O-DF standards for reaching interoperability in a mobile application for the smart city context, in particular for the Smart Mobility domain, electric vehicle (EV) charging case study. The proof-of-concept of the smart EV charging ecosystem with mobile application user interface was developed as a part of an EU (Horizon 2020) Project bIoTope.

## 1. Introduction

Over the last 20 years, the Internet has become widely used in the world [1]. Ubiquitous connectivity, provided by Internet, has enabled the communication paradigm called the Internet of Things (IoT). IoT emerged from domains such as logistics, where it was proposed as a solution to tracking shipments and goods in the supply chain, notably using Radio Frequency Identification (RFID) technology. One of the early descriptions of IoT in the way that we understand it nowadays was in 2002, in the article by Huvio, Grönvall, and Främling [2], who created a lightweight distributed system to share information by using peer-to-peer connections for tracking shipments, but also for accessing any product information during a product’s lifecycle, including sensor readings and other events. Following this definition, IoT allows the development of a huge variety of applications in various industries that will use the enormous quantity of data generated by IoT devices such as cameras, home appliances, sensors, actuators, vehicles, and so on. These applications can allow citizens, companies, and public administrations to benefit in everyday life by using new digital services that are built on top of an IoT ecosystem [3].

Nowadays, many national governments are willing to adopt digital solutions in the management of public affairs, trying to make the concept of the ‘smart city’ a reality [4]. The vision of smart cities includes efficient and sustainable use of urban resources, performing well and in a forward-looking way in economy, people, governance, mobility, environment, and living [5]. Smart cities are seen as a way to mitigate pollution and improve the quality of life of citizens. IoT plays a significant role here, being one of the key enablers, together with Open Data and supported by interoperability between systems [6]. This fosters stimuli to create new services using IoT technologies and ecosystems, where the data created by these services can be used to increase transparency and show the actions of local governments to the citizens [7]. The smart city market is estimated to reach hundreds of billions of dollars by 2020, with annual spending around 16 billion [8].

Of the many domains covered by smart cities, our particular interest is in electric vehicles (EVs) in the context of IoT. Expectations regarding electric vehicles are high, with global sales possibly reaching 5% of the overall light vehicle market by 2020 [9]. EVs are expected to pave the way to more sustainable mobility with zero greenhouse emissions, although the impact will depend on primary energy production methods [10]. Should large scale adoption become reality, city power grid infrastructures will need to be up to the task, with investments made in the actual distribution networks [11]. Combining charging infrastructure with conventional gas stations may not be appropriate here, due to the relatively longer charging process. Hence, researchers have addressed the issue by developing efficient models for charging station placement and optimizing charging scheduling [12,13]. However, large scale penetration of EVs is subject to other significant techno-economic and societal challenges, varying from infrastructural requirements to consumer worries, including safety, reliability, range, battery life, vehicle price, and service availability [14,15]. Furthermore, charging station placement optimization assumes a general availability of all stations to all users. In the real world, the situation is quite the opposite. EV charging services are provided by car manufacturers’ proprietary systems such as Tesla’s Superchargers, or by third party operators, each requiring customers to individually sign up. Such a patchwork of possibly unreliable services can bring frustration to consumers [16].

In general, the smart city domain faces several challenges, where one of the main challenges is non-interoperability of various technologies used in the city and urban development [17,18]. The smart city environment consists of multiple entities that belong to different domains: users, network providers, transport services, buildings, and so on. In addition to it, some of the characteristics of the IoT environment, such as heterogeneity and constrained resources, lead to vertically-siloed IoT applications [19,20]. The data in these applications is usually ‘locked’ in one domain, one system, or one service provider, and cannot be shared and reused by another application, provider, or system. Each system uses its own pack of technologies that are not easily interoperable between each other [21]. This leads to the development of multiple closed vertical systems that generate and use data on their own. To release the full potential of the smart city concept, the cities should be organized as horizontally-oriented open systems in which it is easy to interconnect and exchange data. This will foster the development of new IoT applications that will use the data created mutually by all existing smart city services. 

This article is an extension of the conference paper published in the proceedings of the IEEE Global IoT Summit 2018 [22], the extended content of which included: (i) Deeper discussion of what interoperability is, and which challenges exist on the way of reaching it in the data exchange process between systems. (ii) Introduction of one of the key smart city IoT project in the EU, bIoTope, with a description of the key technologies that form the basis of the project. These technologies, in particular messaging protocols Open Messaging Interface (O-MI) and Open Data Format (O-DF), are the part of The Open Group standardization initiative aimed at providing interoperability for data exchange between various IoT systems. (iii) Presentation of the solution for a Smart EV charging service that was built on top of O-MI/O-DF IoT messaging standards. The contributions of this paper are manifold: (i) It reviewed in a concise way the interoperability issues/challenges in the IoT due to heterogeneity of technology and systems; (ii) proposed to tackle this challenge by using open messaging standards (i.e., O-MI and O-DF), and an IoT marketplace also relying on those standards, for a complete IoT ecosystem that breaks the barriers of vertical data silos and enables horizontal interoperability; and (iii) evaluated this proposition through a real implementation of the bIoTope ecosystem, within the smart city domain. The evaluation, relying on an innovative EV charging use case, was achieved in terms of throughput and response times. This enables conclusions to be drawn on future work.

## 2. System and Data Exchange Interoperability

System interoperability is a growing area of interest in the industrial domain, due to the emerging need to integrate new, legacy systems to expand business capability by utilizing opportunity presented by novel digital information infrastructures. These systems need to be interoperable in order to achieve seamless business integration across organizational boundaries [23] and beyond. It is important to understand interoperability within the context of this research, as multiple domains are at play that need to exchange data seamlessly. The EV charging case study demonstrates that the car’s positioning and routing engine need to communicate with the smart parking and charging infrastructure. Without the presence of open standards and properly thought through data exchange requirements, it is not possible to achieve this integration. The bIoTope research within which this work is positioned attempts to create IoT ecosystems based on open standards that support interoperability across domains. In the given context, researchers working on the domain of interoperability have defined it based on their needs and context. Geraci et al. (1991) define interoperability as “*the ability of two or more systems or components to exchange information and to use the information that has been exchanged*” [24].

Similarly, other researchers/institute have also defined interoperability as:

- *“The ability of a set of communicating entities to (i) exchange specified state data and (ii) operate on that state data according to specified, agreed-upon, operational semantics”* [25].
- *“The ability of systems, units, or forces to provide services to and accept services from other systems, units, or forces, and to use the services so exchanged to enable them to operate effectively together”* [26].

**Figure 1:**
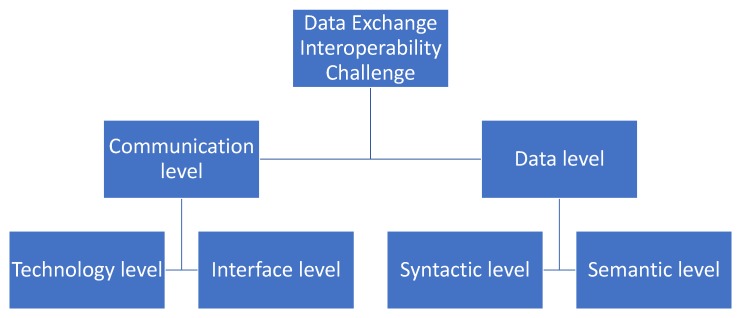
Data exchange interoperability challenge.

Interoperability is a comprehensive paradigm, which can be seen on many levels and in different domain bases. In general, data exchange interoperability can be defined as the ability to communicate, exchange, and process information whenever needed, by whomever needed, with whatever is needed between the two or more entities involved to manage and optimize the objective. Within the given context, data exchange interoperability can be presented as a two-level challenge, as shown in Figure 1.

### 2.1. Communication Level Interoperability

Communication interoperability provides paths, infrastructures, and mechanisms for data exchange between two or more distinct organizations, networks, applications, or IT solutions. In order to achieve communication level interoperability, technological and interface level interoperability need to be achieved.

#### 2.1.1. Technology Level Interoperability

Technology level interoperability is the foundation of data exchange interoperability, which enables two or more systems or entities to communicate with each other. The basic technology level interoperability can be achieved by common agreement of data transmission medium, low-level data encoding, and rules of accessing the medium [27]. Basically, it combines hardware and software, which unifies the physical communication channel and the low-level protocol structure [28]. Since Transmission Control Protocol/Internet Protocol (TCP/IP) is widely is accepted as the solution for technology level interoperability, it can be concluded that technology level interoperability is fully developed [29].

#### 2.1.2. Interface Level Interoperability

Interface level interoperability should provide the necessary communication interface which allows the systems to communicate with each and exchange data or information whenever needed, by whomever needed, and with whatever is needed. These communication interfaces need to be general, and able to cater to the data exchange requirements of each of the systems. To gain time and efficiency, and to avoid re-defining of the co-operation rules and software supporting it each time, these references must be based on business standards or norms. The business standards must be independent and only weakly coupled with the technology, so as to support openness.

### 2.2. Data Level Interoperability

While communication level interoperability enables data exchange between two or more heterogeneous systems, it does not dictate the format and interpretation of the data that has been exchanged. Communication level interoperable systems can be compared to two or more people from different countries speaking their own language to each other, where messages are received but not understood. Hence, data level interoperability should deal with the structure and semantics of data to make systems interoperable.

#### 2.2.1. Syntactic Level Interoperability

Syntactic interoperability refers to the format and structure of data that has been exchanged between heterogeneous systems. In order to achieve syntactic interoperability, data exchanged between systems that are interoperable at communication level should have a well-defined syntax, encoding, and structure for the data or information that are being exchanged. These data formats should be generic enough to be able to represent any data or information that will be exchanged. Syntactic level interoperability allows heterogeneous systems to understand the structure or format of the data, however it does not let the involved party to understand the meaning of exchanged data.

#### 2.2.2. Semantic Level Interoperability

While syntactic interoperability refers to shared format and structure of exchanged data, semantic interoperability deals with the shared meaning of it between heterogeneous systems that are designed for different purposes. The main objective of semantic interoperability is to ensure that the meaning of exchanged data is understood precisely by communicating entities, with the purpose of creating value out of it. Generally, semantic interoperability can be achieved via ontologies that are used to define concepts and relations between these concepts in a certain domain. The ontologies can be linked with data to make logical inferences as to the meaning.

## 3. Overview of bIoTope System

In the following section we will introduce the bIoTope project and the connected smart objects ecosystem. We will also discuss the main building blocks of the ecosystem: O-MI and O-DF messaging standards for IoT, and show how they foster interoperability in the IoT ecosystem.

### 3.1. bIoTope Project and Ecosystem

The bIoTope project is run by several partner organizations in the EU, with the aim to build an open innovation IoT ecosystem for connected smart objects. The project is mainly concentrated upon creation of the platform, based on Fog computing and distributed analytics, using the interoperable API that supports efficient publication and consumption of data from various cross-platform information sources [20].

The bIoTope ecosystem allows the integration of existing data and service providers, such as weather stations, car manufacturers, and so on. At the same time, it allows any organization or private person to publish their personal data or IoT services, and to expose them to the platform using O-MI/O-DF standards for interoperable IoT messaging. The providers of data have a choice of what data to expose, for how long, and at what cost, and are also notified when their data is being consumed and by whom. In this way, the ecosystem will provide a platform that will act like a marketplace, where possible producers of IoT smart services and their consumers will meet and collaborate. The marketplace will offer a payment module and a search engine, which will allow users to search services based on space, keywords, data/service quality/reputation, or technology.

In order to use the services of the marketplace, the third-party developers must agree to the contract terms, then the marketplace sends the API access security tokens that will enable the usage of required services or data from the remote O-MI node/server [20]. O-MI is a generic Open API for RESTful IoT services and systems. O-DF, which is designed to be used paired with O-MI, is a content description model for the Things in the IoT. O-DF can be extended with specific semantic web vocabularies: domain-independent (such as iot.schema.org) and domain-dependent (such as Mobivoc for mobility industries, HL7 for healthcare industry, and so on). In the next sections we provide more details about O-MI/O-DF protocols [20] and on the bIoTope IoT services marketplace IoTBnB.

### 3.2. O-MI: Open API

The Open Group messaging standards emerged from the EU projects PROMISE in FP6 and LinkedDesign in FP7, where the industrial applications collected real data from various products on the instance-level. Because there was no existing standard which could fulfill all the requirements, the group of partner organizations started to develop the specifications of a new messaging standard for IoT. The Open Group messaging interface O-MI is set up as a node, acting as a server and a client. O-MI nodes communicate with each other using interfaces defined in O-MI. As described by Kubler et al. [30], “*the Open Group messaging standards reside in the Application layer of the OSI model, where O-MI is specified in as a Communication level and O-DF is specified in as a Format level. The Open Group standards are used for conveying lifecycle-related information mainly intended for automated processing by information systems, transporting payloads in nearly any format*” [30], such as XML, JSON, CSV, etc., using a variety of low-level transportation protocols from HTTP and SMTP to USB sticks and SMS messages. That provides the technology level interoperability. O-MI provides the interface for peer-to-peer communication, including three basic methods. A main property of O-MI is the concept of deferred retrieval ‘read’ of information, which corresponds to the two available subscription mechanisms: with or without callback address [31]. Other operations of O-MI include ‘write’ to send updated details to the O-MI node, and ‘cancel’ to stop the subscription [32,33,34].

### 3.3. O-DF: Data Model for Things in IoT

Open Data Format (O-DF) is a standard for representation of payload for IoT applications. It was proposed by The Open Group in order to represent information entities about different objects. The representation of these objects in O-DF is general, independent from application or context. O-DF messages can be transported via various methods: by O-MI, via network by different low-level network protocols, or even manually by USB storage drive [35]. 

Designed to provide syntactic data level interoperability, O-DF is specified using XML schema. It is designed to create information structures in a similar way to how they are created in object-oriented programming: objects and properties of objects. It is a generic standard for representation of any object, and it is useful in such domains as IoT and lifecycle information management by overcoming the problem of publication of data from different sources, and filtering the data according to various parameters.

The structure of an O-DF message is a hierarchy with an *Objects* element on top of it. This element can contain any number of *Object* sub-elements. Each *Object* element usually has an id sub-element as an identification, and an optional description sub-element that provides additional information about the object for the users. 

*Object* elements also have properties represented with an *InfoItem* sub-element and any number of *Object* sub-elements. An *InfoItem* sub-element can contain an additional sub-element called *MetaData*, which has a name and values that represent values from the context of *InfoItem*. *MetaData* contains the description of *InfoItem*, though the structure of it is similar to the structure of *InfoItem*. Every *Object*, *Infoitem* element, and even the *value* of each of these elements can have a *type* that can be specified using any semantic vocabulary. It provides semantic level interoperability between IoT systems and specifies the semantic data models. The types of *Objects* or other variables can be set to be related to the system’s domain, in which case the specific vocabularies should be used. It is also possible to model the data using domain independent vocabularies like Schema.org. O-DF offers a way to create, manage, and send information about Things in IoT in a standardized, understandable and universal way. It was designed to be transmitted by O-MI in a query/response format, but is not limited to this transportation method.

### 3.4. bIoTope Marketplace: IoTBnB

One important objective when developing an IoT ecosystem is to provide a way to incentivize its IoT stakeholders (e.g., developers, analysts, businesses, citizens) to join the ecosystem and share (for publishers) and/or consume (for consumers) IoT data/services. To this end, a digital marketplace has been developed for the bIoTope participants. This marketplace—referred as IoTBnB, standing for “IoT service puBlication and Billing” (http://iotbnb.jeremy-robert.fr, last accessed October 2018)—is therefore intended to assist O-MI node owners by making visible their exposed data/services items to the bIoTope ecosystem stakeholders (and eventually making money out of it), and to assist data consumers to search (and pay, if not free) for valuable data/services so as to create new services (such as the smart EV charging case study presented in Section 4) on top of it.

**Figure 2:**
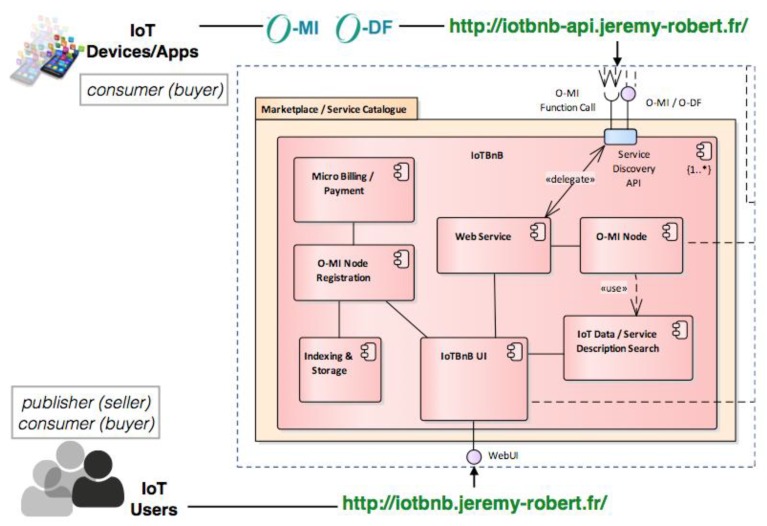
IoTBnB internal components.

IoTBnB consists of two interfaces—a *webUI* and an *API*—and several (internal) components as shown in Figure 2. The IoTBnB UI enables IoT publishers and consumers to access the services offered by the digital marketplace, such as the O-MI node registration (for publishers) and the IoT data/service description search (for consumers).

A publisher wishing to register its O-MI node on IoTBnB provides information about the node itself (e.g., its url, a name, the authentication parameters—if any), thanks to the O-MI node registration UI. Once done, IoTBnB starts the process for collecting, indexing, and storing the set of O-DF InfoItems descriptions (i.e., without the values) exposed by the O-MI node. This component must rely on an appropriate backend data storage system that efficiently manage the storage and indexing of a potentially huge amount of IoT data/service description (i.e., metadata) published by a number of O-MI gateways spread all over the world. To this end, the choice made in bIoTope is to use the platform, offered by our partner OpenDataSoft (https://www.opendatasoft.fr, last accessed October 2018), that already manages metadata of open data in cities. In this context, a specific harvester for O-MI nodes has been developed. If a publisher wants to make money out of it, they have the option to fill out some financial information (such as the address of their wallet, the price of the exposed data/services) thanks to the ‘micro-billing/payment’ module (based on a Bitcoin-based cryptocurrency).

A consumer wishing to have access to available data uses the IoT data/service description search UI, enabling multimodal search like spatial/temporal- or keyword-based search. Let us note that this service is also accessible for IoT devices/apps through the API interface, managed by an O-MI node. This requires use of the O-MI protocol to call this service, as it will be more described in the Section 4.2.

## 4. Proof-Of-Concept: Smart EV Charging

The case that we describe in the current article is part of smart mobility pilot of the bIoTope project for Helsinki region. The smart mobility pilot includes all the services that can be in demand for the car drivers or offered by them: finding free suitable parking lots, finding available charging stations, effective route planning service, publishing information about the car, and making it available for renting out services. We will describe the implementation of the proof-of-concept using O-MI/O-DF messaging standards in the following scenario, primarily focusing on smart EV charging: the EV vehicle arrives in Helsinki and needs to find an available EV charger to recharge the car. In the following sections we will describe the user scenario and user requirements, the implementation of the smart EV charging system, and its mobile user interface (UI) for Android OS (the demo video of the proof-of-concept can be accessed at the following URL https://youtu.be/6DKz1URGamE).

### 4.1. EV Charging Service: Scenario and Requirements

In the presented scenario, the user is the owner of an electric vehicle who enters the city in her car. She needs to recharge the car, therefore she uses the mobile app to search for charging stations. The user requests the list of parking lots with available EV chargers according to the desired destination, or wants to look for the chargers near her current location. The location of available chargers is displayed on the map with related pins. The user is able to save her preferences for the charger, and also sort the search according to charger qualities and for the preferred location. The user sees whether the charger is available or occupied at that moment. The user picks her preferred charger and drives to the charger’s location, where she identifies herself as the user who booked it and starts the charging service [36]. 

Among the main requirements of the system is the accessibility of data in a real time database that contains reliable data about chargers, and shows the availability of the charging points to the user [36]. Different types of data should be available in order to qualify and fit user decisions in the best possible way: detailed location of the charger, availability of the charger at the moment of search, opportunity to reserve the charger in advance, the characteristics of the charger (type of charger and charging adaptor, charging speed), conditions of usage, and payment with price specifications.

**Figure 3:**
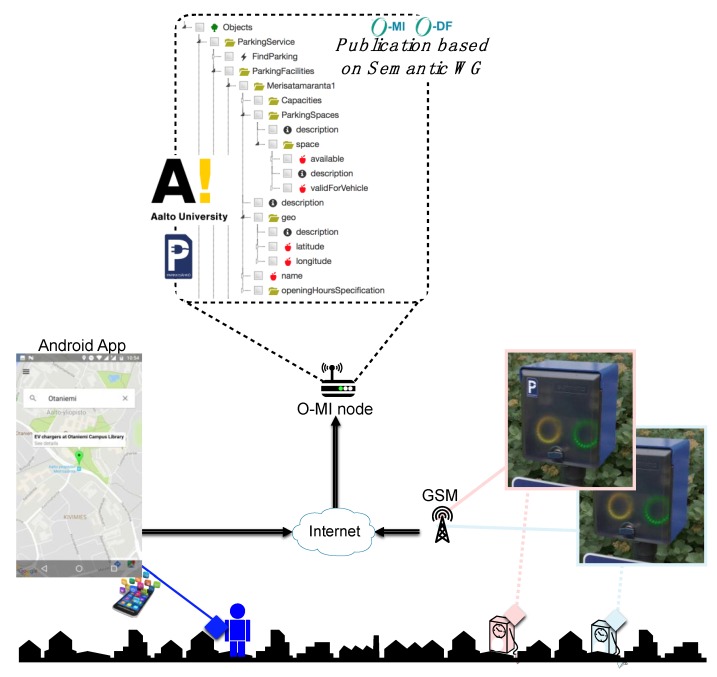
Parking and charging service overview.

The EV parking and charging system consists of three main components (Figure 3). A client, represented by a mobile application for Android OS, is a tool for searching and enabling the EV chargers in the designated area. The backend part consists of several layers with O-MI node in the core. Communication between server and client is done using O-MI/O-DF messaging standards. An O-MI node communicates with the EV charger system provided by bIoTope partner organization Parking Energy, which owns the EV charging poles around Helsinki. Specifically, for this proof-of-concept implementation four EV chargers were installed in the Aalto University parking lot for testing purposes.

**Figure 4:**
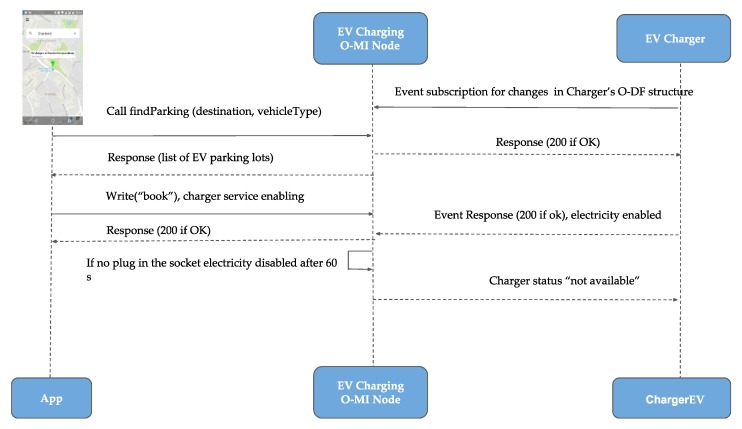
EV charging service sequence diagram.

The general system functionality in the case that the client communicates directly with the chosen O-MI node can be illustrated by these points (Figure 4): Charger creates event subscription to Parking Service for changes in EV Charger’s parking space O-DF structure. Service answers with Response containing returnCode = 200. An electric vehicle enters Helsinki city, and the user sends the geographical query to the mobile application to search for a parking facility with available EV chargers. The detailed view of the user interface interactions can be seen in Figure 4. App sends a *call* request to the specified O-MI node that provides the Parking Service for method “FindParking” with information of destination and type of vehicle, etc.

The Parking and Charging Service (the O-MI node) returns the list of parking facilities with parking spaces containing EV chargers. The user can choose the particular parking space with an EV charger and navigate to it. The route towards the EV parking spot destination can be defined using Google Maps automatically from the app. The user can use the currently available EV parking spot and enable the EV charging service by sending the “book” *write* request to Parking Service specifying the ids of Parking facility, Parking space and Charger id. If Parking and Charging Service approves the reservation it answers with response containing returnCode = 200. The charging service is enabled and there is one minute to put the charging plug into the socket while the electricity flow is running. After the one-minute time period the electricity flow is disabled and the user has to repeat the ‘book’ *write* request. After the Charging service is enabled O-MI node changes Charger’s status to ‘Not available’.

In the following sections, we will separately describe the user interface of the service (an Android application) and the back-end part of the service (O-MI node linked to the EV chargers system of Parking Energy).

Let us note that the app needs to discover the available O-MI nodes publishing EV charging-related services, and in particular the ‘findParking’ method. It requires that O-MI node’s owners register their node(s) on the bIoTope marketplace (IoTBnB). Once done, new, innovative services (such as the app presented in this paper) can be built on top of it using the IoTBnB API to get the O-MI node’s URL expressing such services. More details about this bootstrap phase are given in Section 4.2.

### 4.2. From EV Chargers to Their Discovery through IoTBnB

**Figure 5:**
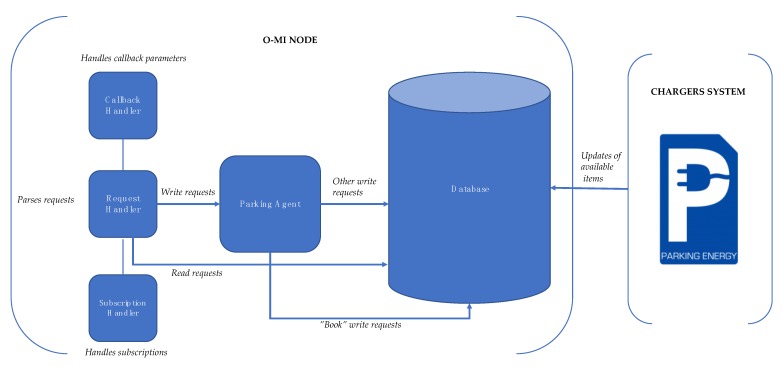
Parking and Charging Service Architecture.

The backend for the EV charging application was implemented by a team of programmers for the bIoTope project. The backend consists of the O-MI node parking and charging service and the EV chargers system of Parking Energy—a partner of bIoTope that provides EV charging services and poles. The O-MI node is set up on the backend of Parking Energy, and is organized over several components that communicate with each other (Figure 5.). The current underlying transport protocols that are supported by the O-MI node are HTTP and websockets. Any O-MI operation is transported using an HTTP POST or websocket text frame.

*O-MI service*. This is the element that is responsible for parsing the requests, authentication and authorization of the client. Each request is handled by the *Request Handler*. The *Request Handler* sends the particular request to the next responsible element, according to the nature of the request. All ‘*read’* requests are sent to the database, which is maintained by the server. The *Database* stores the data about O-DF structure, including *Object(s)* and *InfoItems*. The *Database* periodically gets the availability data from the charger system of Parking Energy, which is used in calculation of capacity of each parking facility item. All requests concerning ‘*write’* operations are distributed by the Request Handler to responsible agents.

*Agents.* Agents are the entities that can programmatically interact with the core of an O-MI node. Generally, an agent is a worker thread that gets data from different data sources separately. For that, it uses specific protocols and turns them into objects that can be used by the O-MI node core. Current implementation has only one *Parking Agent* that communicates with the database, and makes the necessary changes to the data inside the database if the write request contains an update of the parameters of the existing *Object* or *InfoItem*. If there is a ‘book’ request (to use the EV charger) the *Parking Agent* communicates directly to the Parking Energy’s system of chargers and sends the book request to enable the charging service.

*Subscription Handler.* There is a subscription mechanism offered by the O-MI server that allows responses based on the events or time intervals to a number of subscribers. In the current system, there is only one subscriber to the O-MI server database according to the ‘write/call’ requests. It is the external EV charger—a hardware element of the system that aims at receiving notifications about the lid status. All subscriptions and polls are handled by the *Subscription Handler*, which receives related requests from the *Request Handler*. The *Subscription Handler* is also responsible for polling the data using subscription ID integrated into the read request (in cases when a callback address is not provided). In order to eliminate the active subscriptions, the *Subscription Handler* processes ‘*cancel’* requests that stop and delete related subscriptions.

*Callback Handler.* An O-MI server provides subscribers the callback feature, when the subscriber can specify a different URL from the address that has sent the request. This address is called the callback address. The message will then be sent to the callback address during an active subscription. To handle connections and reliable sending of callback messages, there is an element called *Callback* *Handler*.

**Figure 6:**
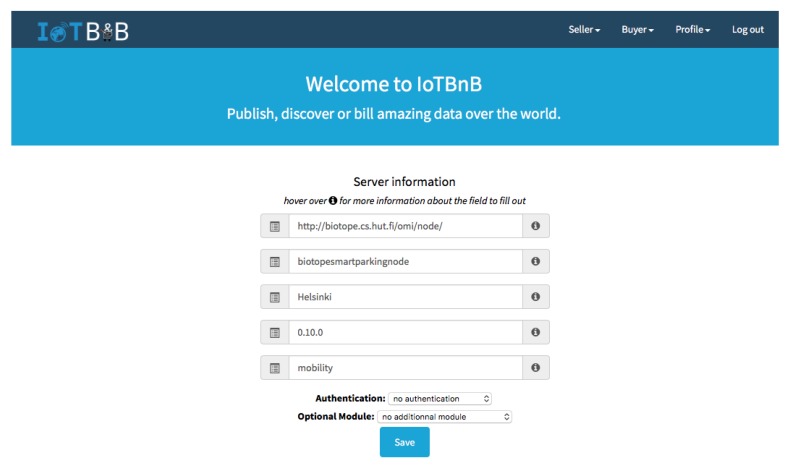
Server information to be filled out by the O-MI owners.

Once an O-MI node is set-up and in operation, its owner can give bIoTope stakeholders the opportunity to discover and use the published data by registering the node on the marketplace IoTBnB. To do so, an Aalto member must be logged in with access to IoTBnB, e.g., by using his/her Google credentials. Then, as the objective is to register his/her node, he/she opens the server information page and fills out the O-MI node-related information, such as the URL of the node (i.e., http://biotope.cs.hut.fi/omi/node/), a name for this node (e.g., biotopesmartparkingnode), the location of the node (i.e., Helsinki in this example), the version (here 0.10.1), a keyword describing most of the exposed data (e.g., mobility), and potential optional modules (such as authentication, billing, or others) as shown in Figure 6. Finally, he/she can save this information. This action sends a request to the indexing and storage component (as described in Section 3.4) to start the process of indexing all the information exposed through this O-MI node. The user can see his/her registered data through the page server → published data.

**Figure 7:**
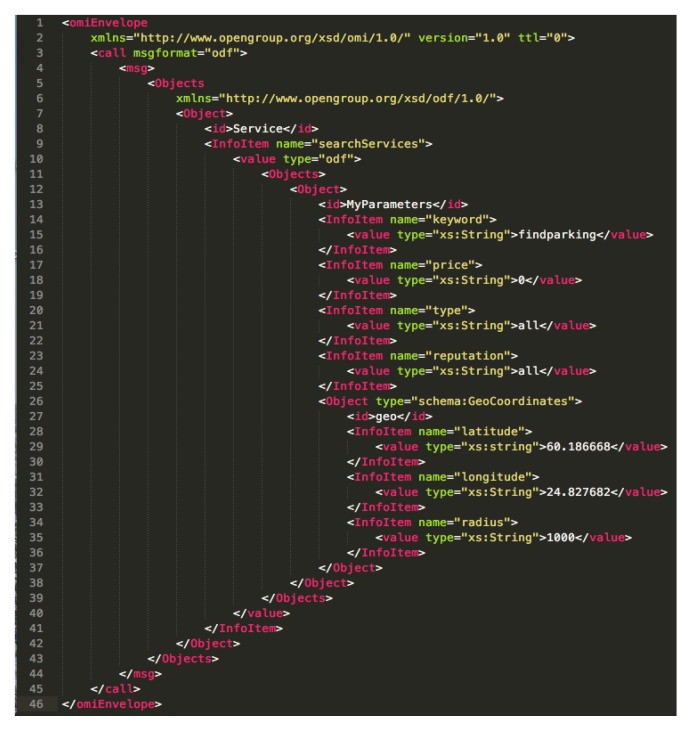
Searching request to IoTBnB API.

At this time (or at least, when O-MI have been registered on IoTBnB in the bootstrap phase), any data consumers and/or apps can use the API of IoTBnB to discover specific O-MI nodes publishing data related to their needs. In this specific case, they would need to send an O-MI/O-DF request to the API URL (which consists of an O-MI node as backend, cf. Section 3.4). The API enables data to be searched according to a keyword, the price, the type (e.g., monitoring, mobility), the reputation score (if any), and also the geo-location (geo-coordinates plus radius). An example of a request is given in Figure 7. It represents a request for searching services according to the keyword ‘findparking’, with a price equal to 0 (i.e., free data), for whatever the type that is defined for those data (i.e., here, type equals to ‘all’), in an area of 1000 m around the coordinates [60.186668;24.826682]. When IoTBnB receives this request, it uses the IoT data/service description search to access the indexing and storage component, so as to retrieve the list of the available services and their parameters (such as the exact location of the node or of the infoItem, and the url to access the service) according to the input parameters, and sends it back to the requester (i.e., the app in this example). Based on this answer, the app is able to call the ‘findparking’ method on all the available O-MI nodes in the area of interest (so as to get the list of the EV parking lots as shown in Figure 3). The next section presents the UI of this app. 

### 4.3. UI for EV Charging Service

**Figure 8:**
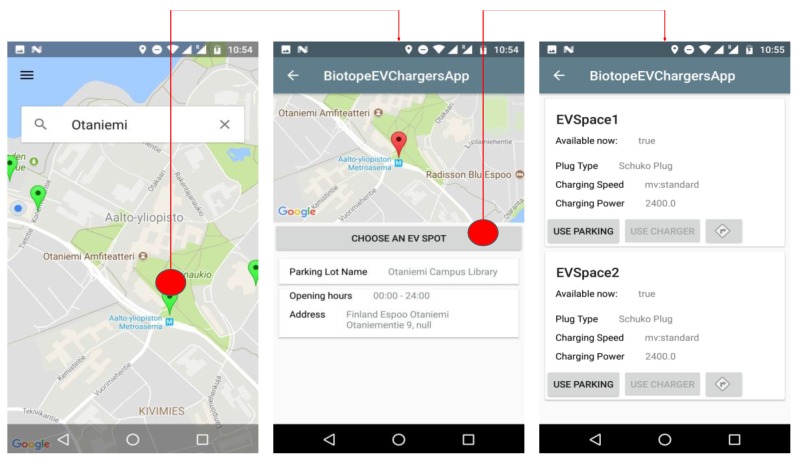
EV charging app UI.

The UI consists of three screens (see Figure 8). The first launcher screen has the Google Map and search bar for the user’s desired location of charging point. The search bar uses the Google Autocomplete service, which guesses the location name and offers variants to the user.

The search results for the desired location will show available EV parking lots pinned on the map. A click on a pin opens the second screen, which provides parking lot details and the opportunity to see the list of EV chargers in each parking spot. By clicking the button “*Choose an EV spot*”, the user opens the list of EV charging spots that are available in the particular parking lot. To use the EV charger, the user should first reserve the parking spot by clicking the button “*Use Parking*”, and then click the button “*Use Charger*” that becomes available after the parking spot has been reserved.

### 4.4. Data Model for EV Charging Service

**Figure 9:**
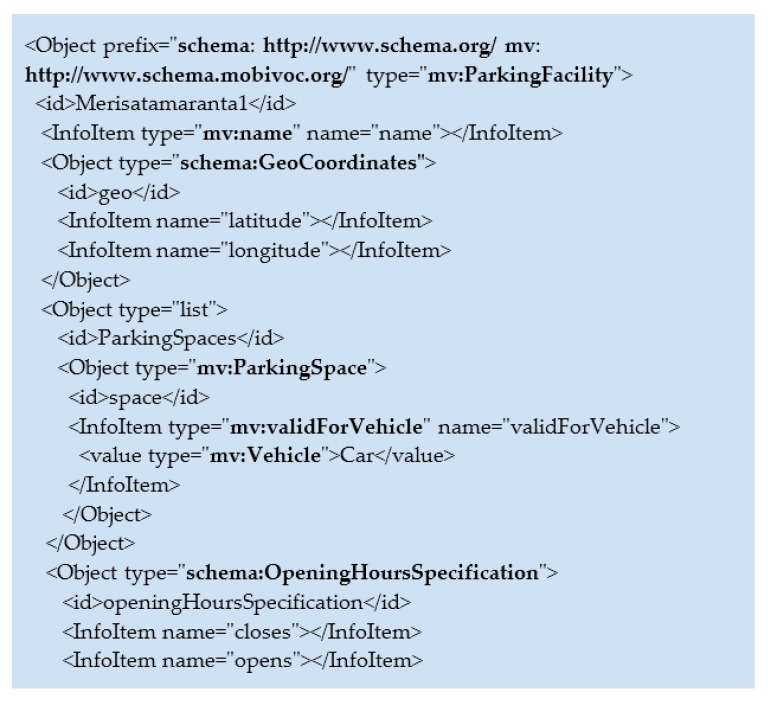
Semantics of O-DF message, *ParkingFacility* object example.5. Performance Evaluation.

The O-DF structure of the parking service is based on semantic data models that provide the semantic enrichment of the objects information. In this case, we chose domain independent Schema.org vocabulary, and Mobivoc vocabulary which is specific for mobility domain data modelling. The example in Figure 9 partially shows the structure of the *Parking Facility* object (a child object of the *Parking Service* object in the general O-DF structure). Two semantic vocabularies are specified in the *Object Prefix* field Throughout the O-DF structure, different fields like *Object type*, *InfoItem type* or *value type* are characterized by each of the semantics vocabularies that provide data level interoperability at the semantics level. By incorporating specialized semantics vocabularies into the O-DF structure, the interoperability is guaranteed by unification of the possible types of data objects. That allows the systems to use the same standardized and agreed-upon types of objects, and provides clear data exchange between services across the IoT platform.

Let us describe the structure of the main object element *ParkingService*. It contains all other nested elements. According to the O-DF structure, *ParkingService* has a list of object elements called *ParkingFacilities* (see example in Figure 9). In its turn, each *ParkingFacility* object in the list has several properties that are represented by the object elements containing several specific InfoItems. As an example, the nested object element *GeoCoordinates* contains InfoItems *longitude* and *latitude*. The *ParkingFacility* object has a list of *ParkingSpace* objects. Each *ParkingSpace* object owns a *Charger* object element that has several InfoItems (*Brand*, *Model*), as well the nested object element *Plug*. *Plug* has InfoItems such as *Power*, *PlugType, Voltage*, *CableAvailable*, *ChargingSpeed*, and *LockerAvailable*. All of these specifications are necessary to comply with the reality of how the EV Charging industry is organized.

The previously presented smart parking and EV charging example was considered mainly to assess the performance of the O-MI nodes used. More concretely, the objective was to evaluate the feasibility of handling several users in a short period of time. We assumed the following scenario: several users (the number varied according to the experiments), using the same Android app, search for the same (or approximately the same) destination in a short period of time. According to the aforementioned behavior, each app will send a request to IoTBnB (and in particular the O-MI node-based API) to get a list of O-MI nodes publishing parking-related information. Based on the response, the app will call the function for getting (and potentially booking) the available parking lots ‘findParking’ (available on the O-MI nodes included in the response).

The O-MI node behind the marketplace IoTBnB (referred as *node1* hereafter) is intended to retrieve an information description on the indexing and storage platform (i.e., OpenDataSoft as cited previously) each time it receives an O-MI/O-DF request, and convert the related data into an O-DF payload to send back to the user/app. This O-MI node (version 0.9.2 of the reference implementation) is currently running on a virtual machine with the following features: (i) CPU: 4 cores @2.4 GHz (ii) operating system: Ubuntu 14+ and (iii) memory: 4 GB of RAM. It is hosted behind a firewall on the University of Luxembourg network. The second O-MI node (referred as *node2* hereafter), hosted at http://veivi.parkkis.com:8080, is owned by a bIoTope partner (Parking Energy) that manages parking lots in Helsinki region, whose information is published through this node. The objective was to perform a (stress) test to observe the throughput that these O-MI nodes can handle. Note that both nodes were assessed independently, and a comparison was also achieved to demonstrate the improvements in terms of performance between old and most recent versions of the O-MI reference implementation. Apache JMeter11 (available online at https://jmeter.apache.org) enabled the simulation of multiple users requesting the O-MI nodes. This open-source software was run on a MAC Book Pro Retina (mi-2015) with a CPU Intel Core i7 2.8GHz and memory of 16GB 1600MHz DDR3.

**Figure 10:**
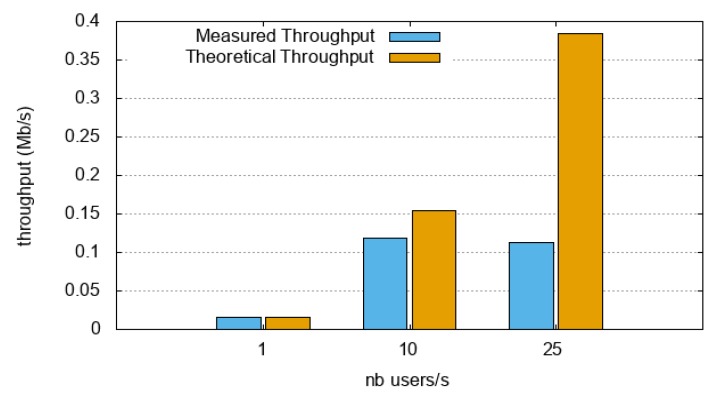
Throughput evaluation under stress/load test on node1 (IoTBnB O-MI node).

**Figure 11:**
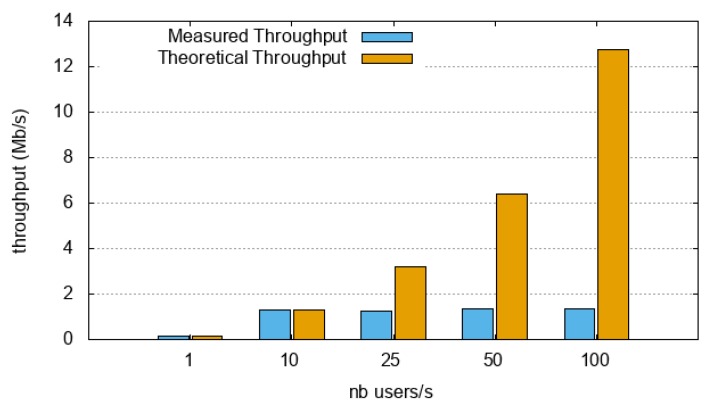
Throughput evaluation under stress/load test on node2 (Parking Energy O-MI node).

First, we evaluated the maximum (measured) throughput that the O-MI nodes could handle. To do so, thanks to the JMeter software, 1000 concurrent users were created that sent only one time respectively (i) the search request depicted in Figure 10 for the IoTBnB O-MI node, and (ii) a call request (of the ‘findParking’ method) for the Parking Energy O-MI node. The experiment was carried out many times with 1 user, 10 users, 100 users, and so on. The more users created per second, the more concurrent users and load was put on the O-MI node. Results are given in Figure 10 and Figure 11.

Based on the resulting throughput evaluation (cf. Figure 10 and Figure 11), the following conclusions can be drawn:

- *node1* was easily able to manage the load when 1 user/s was created (i.e., a throughput equal to 0.013 Mb/s); as each user generated only one request, the server needed to handle only one frame per user at a time (i.e., a traffic load—request + response—equal to 1924 bytes). When the number of users was increased to 10 and 25 users/s, the measured throughput was approximately 0.03 Mb/s, that is, less than the theoretical throughput (around 0.15 Mb/s). In addition, the error rate was significant with 10 users/s, and 100% with 25 users/s. In those specific cases, the server was not able to manage the load. This could be explained by the fact that (i) the server could not manage the backlog (this especially might have been the case, since the server needed to send another HTTP request to the OpenDataSoft platform), (ii) the service could have been unavailable at that time, or even (iii) the university infrastructure could have limited the authorized bandwidth. Overall, *node1* was able to manage a maximum throughput of around 0.013 Mb/s. Note that the version of the node itself is an old version, which is not even available on Github (version 0.9.2).
- *node2* was able to manage the load with a user creation rate of 1 and 10 users/s (i.e., a throughput equal to 0.13 and 1.3 Mb/s). Beyond that (i.e., with 25, 50 and 100 users/s), the measured throughput was stagnant at the same value. This means that the maximum throughput this O-MI node can handle is around 1.3 Mb/s. Let us note that in these specific cases, (i) the error rate was very low (less than 1% at 25 users/s, and less than 7% beyond), and (ii) the total traffic load was 15966 bytes (more than 13,000 bytes to retrieve and to send back in the response), i.e., 8 times higher than in *node1*.

In comparing both nodes and implementation, one can see that *node2* could more easily manage the load than *node1*. The same conclusion was drawn with an older version (v0.6) on a resource-constrained device (i.e., a Raspberry), where the response time increased a lot when the load increased (scenario was concurrent users generating unlimited requests), and in that particular case, 30 users was somehow a maximum [37]. However, despite these current limitations, one can see the continuous improvements of the development team, enabling better and better performance throughout the project. 

We continued the experimental evaluation by considering a scenario where each O-MI node was still able to respond to the requests (but relatively near to the assessed limit, i.e., with a throughput of around 0.013 Mb/s and 1.3 Mb/s users/s as aforementioned). We assessed the response times by varying the total number of users (10, 100 and 1000) while keeping the creation frequency at 10 users/s. It enabled us to assess how the server was able to assess the backlog. Results are given in Table 1 in terms of minimum, mean, standard deviation, and maximum response time.

Both O-MI nodes gave relevant results for such applications, where the load was under the throughput limit of the nodes. Let us note that we considered as relevant was a response time under 1s to give a good quality of experience when using the EV charging app. However, one may note than response time could reach 3 s (or 10 s) in the worst cases, due to either a network delay or a server backlog. As we are on the Internet, some message losses can occur sometimes, but in our experiments, thanks to the TCP protocol (that can manage retransmission if needed), no losses happened.

Overall, the stated goal was to conceive a proof-of-concept implementation to show how O-MI/O-DF standards can be used for solving interoperability issues, and there were no needs at that time to manage a heavy load and a lot of users. In that sense, all the results are, at this stage, satisfactory. This is all the truer since our experiments showed that the new version of the O-MI node is improved in terms of performance compared to the older one. In a long-term vision, it is worth pointing out that the load will be distributed across different O-MI nodes (meaning that all parking lots of one city could be published by several nodes, since they would be owned by different business parties) some of which could even be redundant if needed. Regarding the marketplace, even if one can imagine that more than many thousands of users will access the marketplace regularly in a short period of time, additional mechanisms can easily be implemented to be fully scalable if needed (e.g., when a business partner takes the lead on development and makes a business out of it). This kind of heavy load can be handled, as with any website, by using load balancer systems and redundant and/or distributed instances.

## 5. Conclusions

The EV charging example demonstrates that it is possible to create distributed, bottom-up services, based on open standards, that can contribute to the smart cities ecosystem. The proposed approach can be extended to many other practical applications, for example to monitor empty bike parking spaces, to search for local peer-to-peer services, monitor garbage trucks, safe routing services for school children, etc. IoTBnB can become a peer-to-peer market place for the bottom-up innovation that the smart cities domain clearly needs.

IoT technology allows millions of devices and smart objects to communicate and share data with each other. However, the data is stuck in vertically-oriented closed systems with limited interoperability between the system blocks. This creates a hindrance for the development of IoT markets, and limits the connection between the various participants in IoT ecosystems. This issue is especially important in the smart city context, where the ecosystem comprises many different service providers that must collaborate efficiently with each other in order to ship high quality services to the citizens. This article discussed the opportunity of horizontal data exchange within the IoT ecosystem, using IoT messaging standards O-MI/O-DF that were published by the Open Group mainly for back-end data sharing. These standards support integration of contextual data from semantic vocabularies of many different domains, which opens opportunities to create more advanced smart services. By providing the description of the proof-of-concept implementation of the smart EV charging case study within the ongoing EU project bIoTope, we demonstrated how the mentioned messaging standards can contribute towards the creation of an open IoT ecosystem with horizontal communication model between the ecosystem’s parts.

Future research can be made on the security and privacy of the open IoT ecosystem, in order to protect service providers and users from harmful and malicious attacks on their data and operations while using the open IoT platform services. In addition, it would be interesting to lead thorough comparison analysis between all existing messaging protocols designed for IoT communications (e.g., Data Distribution Service (DDS) or XMPP protocols, which are also designed for server-to-server communications as are O-MI protocols) and other similar initiatives (like Smart-M3 project), which were beyond the scope of this paper. Comparing Open Group IoT messaging protocols (their ontology-based features in particular) with other ontology-based mechanisms (such as the aforementioned Smart-M3 project) is also an interesting research axis that could be explored in a near future work.

## Figures and Tables

**Table 1:** Response time.

Total Users	Node 1				Node 2			
	Min	Mean	Std	Max	Min	Mean	Std	Max
10	262	333	104	655	383	508	68	893
100	257	298	58	956	498	545	171	3496
1000	253	301	80	2286	210	580	424	10,256
